# Supplementary material for: Identification of BOLD engine deficiencies and suggestions for improvement based on a curated Tachina (Diptera) record set
Source: PLoS One. 2025 Aug 29;20(8):e0331216. doi: 10.1371/journal.pone.0331216 (PMC12396684; doi:10.1371/journal.pone.0331216)
Supplement: Attachment — Submitted filename: PONE-D-25-31493 fpo.pdf [file pone.0331216.s003.pdf]

PLOS ONE

Identification of BOLD engine deficiencies and suggestions for the improvement based on a curated record set

--Manuscript Draft--

**Manuscript Number:**	PONE-D-25-31493
**Article Type:**	Research Article
**Full Title:**	Identification of BOLD engine deficiencies and suggestions for the improvement based on a curated record set
**Short Title:**	Identify and address BOLD engine deficiencies
**Corresponding Author:**	Frederik Stein, MSc<br>Julius Kuhn-Institut Braunschweig Messeweg<br>Braunschweig, GERMANY
**Keywords:**	BOLD; BINs; DNA Barcoding; Species determination algorithm; ASAP; RESL
**Abstract:**	The increasing number of Barcode of Life Database (BOLD) records per species and genus leads to contradictory species assignments within Barcode Index Numbers (BINs), serving as identifiers for the BOLD ID engine. To examine these issues, we analyzed a dataset comprising original and curated BOLD records for the genus Tachina (Insecta, Tachinidae), which was based on a previous publication. This dataset included both published and private records. We were able to assess the performance of the BOLD engine's species determination algorithm, Refined Single Linkage (RESL), and compare it to Assemble Species by Automatic Partitioning (ASAP). Additionally, we investigated the usage of BINs by the BOLD v4 ID engine. Our analysis confirmed that BOLD queries primarily rely on BINs for species identification, even though some cases deviated from this pattern, producing species matches inconsistent with BIN species assignments. ASAP was found to be superior to RESL due to RESL's adherence to the concept of the DNA barcoding gap. Moreover, we found that taxonomic misassignments, inconsistencies in BIN formation, and missing metadata also contribute significantly to unreliable identifications. These problems appear to stem from both algorithmic limitations and deficiencies in submission and post-submission processes.<br>Moreover, we noted that the default mode of the BOLD v4 ID engine integrates both private and published data, leading to public records based solely on COI-based identifications. However, this issue may now be mitigated, as the BOLD v5 ID engine default mode exclusively employs published data.<br>To enhance BOLD's reliability, we propose improvements to submission and post-submission processes. Without such amendments, the accumulation of contradictory species assignments within BINs will continue to rise and the reliability of specimen identification by BOLD will decrease.
**Order of Authors:**	Frederik Stein, MSc
	Oliver Gailing
**Additional Information:**	
**Question**	**Response**
**Financial Disclosure**<br><br>Enter a financial disclosure statement that describes the sources of funding for the work included in this submission. Review the submission guidelines for detailed requirements. View published research articles from *PLOS ONE* for specific examples.	Yes

Powered by Editorial Manager® and ProduXion Manager® from Aries Systems Corporation

This statement is required for submission and **will appear in the published article** if the submission is accepted. Please make sure it is accurate.<br><br>**Funded studies**<br>Enter a statement with the following details:<br>• Initials of the authors who received each award<br>• Grant numbers awarded to each author<br>• The full name of each funder<br>• URL of each funder website<br>• Did the sponsors or funders play any role in the study design, data collection and analysis, decision to publish, or preparation of the manuscript?<br><br>Did you receive funding for this work?	
Please add funding details.<br>as follow-up to "**Financial Disclosure**<br><br>Enter a financial disclosure statement that describes the sources of funding for the work included in this submission. Review the submission guidelines for detailed requirements. View published research articles from *PLOS ONE* for specific examples.<br><br>This statement is required for submission and **will appear in the published article** if the submission is accepted. Please make sure it is accurate.<br><br>**Funded studies**<br>Enter a statement with the following details:<br>• Initials of the authors who received each award<br>• Grant numbers awarded to each author<br>• The full name of each funder<br>• URL of each funder website<br>• Did the sponsors or funders play any role in the study design, data collection and analysis, decision to publish, or preparation of the manuscript?	Project: Risk Management for Biotic Damage to Ensure Sustainable Forest Management (RiMa; FKZ 22019814) funded by the Federal Ministry of Food and Agriculture/ FNR

Powered by Editorial Manager® and ProduXion Manager® from Aries Systems Corporation

Did you receive funding for this work?"	
Please select the country of your main research funder (please select carefully as in some cases this is used in fee calculation).<br>as follow-up to "**Financial Disclosure**<br><br>Enter a financial disclosure statement that describes the sources of funding for the work included in this submission. Review the submission guidelines for detailed requirements. View published research articles from *PLOS ONE* for specific examples.<br><br>This statement is required for submission and **will appear in the published article** if the submission is accepted. Please make sure it is accurate.<br><br>**Funded studies**<br>Enter a statement with the following details:<br>• Initials of the authors who received each award<br>• Grant numbers awarded to each author<br>• The full name of each funder<br>• URL of each funder website<br>• Did the sponsors or funders play any role in the study design, data collection and analysis, decision to publish, or preparation of the manuscript?<br><br>Did you receive funding for this work?"	GERMANY - DE
**Competing Interests**<br><br>Use the instructions below to enter a competing interest statement for this submission. On behalf of all authors, disclose any competing interests that could be perceived to bias this work—acknowledging all financial support and any other relevant financial or non-financial competing interests.<br><br>This statement is required for submission and **will appear in the published article** if	The funders had no role in the design of the study; in the collection, analyses, or interpretation of data; in the writing of the manuscript; or in the decision to publish the results. The authors declare no conflicts of interest.

the submission is accepted. Please make sure it is accurate and that any funding sources listed in your Funding Information later in the submission form are also declared in your Financial Disclosure statement.<br><br>View published research articles from *PLOS ONE* for specific examples.<br><br>**NO authors have competing interests**<br><br>Enter: *The authors have declared that no competing interests exist.*<br><br>**Authors with competing interests**<br><br>Enter competing interest details beginning with this statement:<br><br>*I have read the journal's policy and the authors of this manuscript have the following competing interests: [insert competing interests here]*<br><br>* typeset	
**Ethics Statement**<br><br>Enter an ethics statement for this submission. This statement is required if the study involved:<br><br>• Human participants<br>• Human specimens or tissue<br>• Vertebrate animals or cephalopods<br>• Vertebrate embryos or tissues<br>• Field research<br><br>Write "N/A" if the submission does not require an ethics statement.<br><br>General guidance is provided below. Consult the submission guidelines for detailed instructions. **Make sure that all information entered here is included in the Methods section of the manuscript.**	This study did not involve human participants, human specimens or tissue, vertebrate animals or cephalopods, vertebrate embryos or tissues, or field research requiring permits.

**Format for specific study types**<br><br>**Human Subject Research (involving human participants and/or tissue)**<br>• Give the name of the institutional review board or ethics committee that approved the study<br>• Include the approval number and/or a statement indicating approval of this research<br>• Indicate the form of consent obtained (written/oral) or the reason that consent was not obtained (e.g. the data were analyzed anonymously)<br><br>**Animal Research (involving vertebrate animals, embryos or tissues)**<br>• Provide the name of the Institutional Animal Care and Use Committee (IACUC) or other relevant ethics board that reviewed the study protocol, and indicate whether they approved this research or granted a formal waiver of ethical approval<br>• Include an approval number if one was obtained<br>• If the study involved *non-human primates*, add *additional details* about animal welfare and steps taken to ameliorate suffering<br>• If anesthesia, euthanasia, or any kind of animal sacrifice is part of the study, include briefly which substances and/or methods were applied<br><br>**Field Research**<br><br>Include the following details if this study involves the collection of plant, animal, or other materials from a natural setting:<br>• Field permit number<br>• Name of the institution or relevant body that granted permission	
**Data Availability**<br><br>Authors are required to make all data underlying the findings described fully available, without restriction, and from the time of publication. PLOS allows rare exceptions to address legal and ethical concerns. See the PLOS Data Policy and FAQ for detailed information.	Yes - all data are fully available without restriction

A Data Availability Statement describing where the data can be found is required at submission. Your answers to this question constitute the Data Availability Statement and **will be published in the article**, if accepted.<br><br>**Important:** Stating 'data available on request from the author' is not sufficient. If your data are only available upon request, select 'No' for the first question and explain your exceptional situation in the text box.<br><br>Do the authors confirm that all data underlying the findings described in their manuscript are fully available without restriction?	
**Describe where the data may be found in full sentences. If you are copying our sample text, replace any instances of XXX with the appropriate details.**<br><br>• If the data are **held or will be held in a public repository**, include URLs, accession numbers or DOIs. If this information will only be available after acceptance, indicate this by ticking the box below. For example: *All XXX files are available from the XXX database (accession number(s) XXX, XXX.*).<br>• If the data are all contained **within the manuscript and/or Supporting Information files**, enter the following: *All relevant data are within the manuscript and its Supporting Information files.*<br>• If neither of these applies but you are able to provide **details of access elsewhere**, with or without limitations, please do so. For example:<br><br>*Data cannot be shared publicly because of [XXX]. Data are available from the XXX Institutional Data Access / Ethics Committee (contact via XXX) for researchers who meet the criteria for access to confidential data.*<br><br>*The data underlying the results presented in the study are available from (include the name of the third party*	All data underlying the findings of this study are available in the supplementary data files provided with the following publication: Stein F, Gailing O, Moura CCM. Curating BOLD records via Bayesian phylogenetic assignments enables harmonization of regional subgeneric classifications and cryptic species detection within the genus Tachina (Diptera: Tachinidae). Annals of the Entomological Society of America. 2024:245-256. doi: 10.1093/aesa/saae018.

and contact information or URL).<br>• This text is appropriate if the data are owned by a third party and authors do not have permission to share the data.<br><br>* typeset	
Additional data availability information:	

Click here to access/download;Manuscript;Stein_Gailing_PLOS_one_Manus

Title: Identification of BOLD engine deficiencies and suggestions for the improvement based on a curated record set

Short running title: Identify and address BOLD engine deficiencies

Authors: Frederik Stein[1,2*], Oliver Gailing[3]

[1]: Julius Kühn-Institute, Federal Research Centre for Cultivated Plants, Institute for Forest Protection

[2]: Julius Kühn-Institute, Federal Research Centre for Cultivated Plants, Institute of National and International Plant Health

[3]: University of Göttingen, Forest Genetics and Forest Tree Breeding

* Corresponding author:

Frederik Stein

Email: Frederik.stein@julius-kuehn.de

Abstract

The increasing number of Barcode of Life Database (BOLD) records per species and genus leads to contradictory species assignments within Barcode Index Numbers (BINs), serving as identifiers for the BOLD ID engine. To examine these issues, we analyzed a dataset comprising original and curated BOLD records for the genus *Tachina* (Insecta, Tachinidae), which was based on a previous publication. This dataset included both published and private records. We were able to assess the performance of the BOLD engine's species determination algorithm, Refined Single Linkage (RESL), and compare it to Assemble Species by Automatic Partitioning (ASAP). Additionally, we investigated the usage of BINs by the BOLD v4 ID engine.

Our analysis confirmed that BOLD queries primarily rely on BINs for species identification, even though some cases deviated from this pattern, producing species matches inconsistent with BIN species assignments. ASAP was found to be superior to RESL due to RESL's adherence to the concept of the DNA barcoding gap. Moreover, we found that taxonomic misassignments, inconsistencies in BIN formation, and missing metadata also contribute significantly to unreliable identifications. These problems appear to stem from both algorithmic limitations and deficiencies in submission and post-submission processes.

Moreover, we noted that the default mode of the BOLD v4 ID engine integrates both private and published data, leading to public records based solely on COI-based identifications. However, this issue may now be mitigated, as the BOLD v5 ID engine default mode exclusively employs published data.

To enhance BOLD's reliability, we propose improvements to submission and post-submission processes. Without such amendments, the accumulation of contradictory species assignments within BINs will continue to rise and the reliability of specimen identification by BOLD will decrease.

Keywords: BOLD, BINs, DNA Barcoding, Species determination algorithm, ASAP, RESL

Introduction

The increasing record number per species and the BOLD identifier Barcode Index Number (BIN) [1], driven by the growth of databases and the rising demand for DNA-based biodiversity inventories, highlights the critical need for accurate BOLD reference library entries, the largest DNA barcode database [2]. Since species identities shall uncover the ecological function of individuals, a reliable identification of the described species is essential. Especially, research in plant protection demands this reliability to reveal host parasitoid relations [3], and to identify and to classify pests [2], quarantine pests [4] or their antagonists [5].

Particularly, concerning Metazoa and the commonly used mitochondrial cytochrome oxidase subunit 1 5' (COI-5P) region, challenges in species identification and efforts to curate the BOLD database have been discussed. For example, previous work [1] addressed the issue of screening in submitted sequences for *Wolbachia* spp., bacteria associated with many arthropod taxa as symbiont [6] and parasite [7]. OTU pipeline quality criteria such as a minimal sequence length of 500 base pairs (bp) and less than one percent ambiguous nucleotides have been introduced BINs [1]. These measures ensure adequate sequence coverage [1] and consequently the discrimination of sequences based on sufficient variable sites. Furthermore, when BOLD queries do not match any sequence, the possibility that the reverse complement sequence was entered is considered. Therefore, a tool was provided to generate the forward orientation of the reverse complement sequence [8]. Recently, previous work demonstrated that Bayesian phylogenetic analysis can be employed to curate BOLD data, provided the dataset contains all contradictory species assignments [9].

However, previous work [10] criticized some aspects of BOLD in the frame of a re-analysis of an earlier study [11]. The study [11] provided first species descriptions exclusively based on consensus sequences and BIN partitions. The primary concerns arose by the fact that BINs are generated using the "Refined Single Linkage" (RESL) algorithm, which incorporates both published and private sequences in BOLD [10]. Consequently, this raises doubts about the transparency of BINs as reliable references for specimen identification. Furthermore, BIN formation process is not reproducible since private

sequences are not accessible to users. This has led to critical views on the upcoming tendency to generate new species descriptions solely based on COI-5P sequences [11] and their automatic partitioning into species identities [10] by RESL analysis, which serve as identifiers in BOLD search queries [1]. Furthermore, the documentation of morphological specimen identification in BOLD shall be improved, for example by a mandatory reference annotation for species identification like species keys [12].

We employed a BOLD record set of *Tachina* (Meigen, 1803) representing all contradictory species assignments within BINs up to the date of download [9]. In this study, we employed the resulting complete and curated dataset to compare the performance of the species determination algorithm RESL [1] with the more recent species determination algorithm "Assemble Species by Automatic partitioning" (ASAP) [13]. Additionally, we tested the BOLD v4 ID engine with query sequences representing BINs and species assignments of the dataset. We aimed to evaluate the consistency of results referring to the BIN based BOLD ID engine [1]. In addition, we tested whether records with species assignments based on morphological identification are always included in BOLD queries based on the "Public Records Barcode Database". Moreover, we identified errors and gaps in BOLD metadata, like taxonomically incorrect assignments, questionable species assignments and BIN registries. Beyond, we provide a list of possible actions and procedures to avoid an accumulation of contradictory species assignments in BINs.

We note that the current study was carried our using BOLD v4 and that BOLD v5 was launched 2024. However, no publication or manual has yet been released. We point out that the results page of the BOLD ID engine has been changed, but the former BOLD v4 ID engine can still be used in BOLD v5. Many processes, such as the submission process are still based on BOLD v4 and BINs based on RESL still serve as identifiers. We also note that the BIN system is dynamic, as the BIN registry is updated monthly [14]. In addition, records assigned to *Tachina* may have been added or deleted, since we conducted our analysis. However, as we consider the problems identified in BOLD to be systematic, we assume that they are still present, regardless of the recentness of the employed data set.

Material and methods

Data

For the study, we utilized BOLD data related to the genus *Tachina* with 812 BOLD records. First, the dataset, containing all existing species assignments per BIN (including all contradictory species assignments within BINs) and almost all private and published *Tachina* records, was curated based on a Bayesian phylogenetic analysis of COI-5P sequences from BOLD v4 [9]. Additionally, we modified the metadata by updating BIN assignments in metadata on the 4th September 2023 (Table S1). To identify errors in taxonomical assignments we generated two species assignment columns to the dataset. One column contained species names annotated in BOLD metadata and the second one contained the revised final species names based on curated records [9]. The final species assignments were based on metadata and coherence between the phylogenetic inference and the taxonomic position at the subgenus level. However, we did not revise inconsistent species assignments within subgenera because discrepancies might be due to incomplete lineage sorting, undetected species complexes and horizontal gene transfer mediated by *Wolbachia* bacteria, in addition to technical issues (e.g., misidentification, PCR contamination) [9].

According to the final species assignment, the dataset contained 21 described species, where one species was separated into two cryptic species. Furthermore, there were at least six further species identities labelled with OTU names [9]. One of these OTU names, *Tachina* nr. *magnicornis*, comprised a group of individuals considered to represent one morphologically distinguishable taxon. Three of the six OTU names were allocated to species identities detected with the ASAP algorithm. For the remaining two OTU names in BOLD metadata, we found no further information as to why an OTU name was assigned [9]. The metadata was processed using R 4.3.0 [15] in RStudio 2023.6.2.561 [16] and the package tidyverse [17].

Comparison of species determination algorithms

To compare the performance of the RESL and ASAP algorithms we compared species partitions with the final species assignments [9]. Therefore, we visualized the BIN and ASAP species partitions in the final phylogenetic tree (Fig 1). Information on the allocation of BIN and ASAP partitions to records can additionally be found in Table S 1. For the visualization, we modified collapsed sequence groups to annotate BINs and ASAP partitions. We employed the interactive tree of Life v6 (iTOL) and the iTOL annotation editor v.1.4 [18] to modify the final tree.

BOLD query testing

Furthermore, we employed the record dataset to test the default mode of the BOLD v4 ID engine, to examine, whether the suggested species matches on the results page corresponded to the BIN. In our experience results appear to deviate from this pattern in some cases. We ran default BOLD queries with a set of sequences representing different BINs and species and also employed sequences that were assigned to species but not to BINs. For each BIN and originally annotated and later revised species name we used the longest sequence with the lowest percentage of ambiguous nucleotides.

Examination of publication state and annotated identification method

Moreover, the BOLD ID engine provides an option to query exclusively within the "Public Record Barcode Database," which suggests a higher degree of reliability by limiting results to published records. However, some BINs within this database, such as BOLD:AAG2170, contain only COI-based species identifications without corresponding published morphological validation. Consequently, there is a dependence on private morphological references, which is inconsistent with the expected degree of curation. Therefore, we utilized the sequence assigned to process ID "CNCTS095-18", BIN BOLD:AAG2170 and the species *Tachina nitida* (van der Wulp, 1882) to test the "Public Record Barcode

Database" option of the BOLD ID engine. Furthermore, we visualized how the annotations for identification methods (morphological, COI-based, unknown) are distributed across the dataset according to the publication state.

Identification and classification of errors and gaps in BOLD annotations

The documentation of errors and gaps in annotations were quantified based on counted records, which were affected by concerned errors and gaps (Table 1). We counted records with contradictory species assignments between annotated and revised species names [9]. Moreover, we categorized and identified incorrect taxonomical assignments due to the utilization of outdated species names, incorrect genus assignments, incorrect species assignments, the utilization of subgenus names instead of genus names and outdated COI-based identifications based on the revised species assignments [9]. Outdated COI-based identifications were due to additional record uploads causing contradictory species assignments within BINs. Furthermore, we identified records assigned to BINs but not to species, even though other records linked to the same BIN were assigned to a single species. Since these records would have enabled a species assignment for those records lacking such assignments, we categorized this as "missing species assignments".

Furthermore, we counted records associated with "questionable species assignments". We refer here to contradictory species assignments in species clades of the final tree which hamper unambiguous species discrimination, as it remained unclear whether inconsistencies were due to human errors or species introgression and incomplete lineage sorting [9]. Therefore, we registered records representing species assignments, which were in the minority in concerned species clades as "due to misidentification or species introgression". Records that we identified to produce contradictory information due to too recent speciation events, were not considered, since contradictory information was presumably due to a low resolution of the COI-5P gene locus and not to human errors [9]. A second documented category was questionable species assignments "due to unknown identification method".

This concerns records, which contribute to contradictory species assignments within species clades. Depending on the identification method the concerned records had to be classified as "due to misidentification or introgression" within the same erroneous class or "due to an outdated COI-based identification" within in the erroneous class "incorrect taxonomical assignment".

When sequences were assigned to a BIN conflicting with the BOLD OTU pipeline quality criteria, which require a minimum sequence length of 500 bp and a maximum of one percent ambiguous nucleotides [1], we noted this as "conflicting BIN assignment" within the class "questionable BIN registry". Furthermore, we documented records, which were allocated to a BIN that incorporated at least two species conflicting with the final species assignments and ASAP partitions [9]. The third category error of "questionable BIN registry" included records with paraphyletic species identity.

Moreover, we counted records associated with gaps and errors in the class further metadata. Further metadata was considered as metadata not directly related to taxonomical assignments or BIN registry. Consequently, this class contained deficiencies in metadata like missing information on geographical origin or sex. We documented records, where despite morphological identification, information on sex was missing, as sex determination is part of morphological identification in Tachinidae. Moreover, morphological identification of female Tachinidae is challenging due to less distinct morphological characters available to identify the species [19,20]. Consequently, the information could have contributed to assess the reliability of morphological identification [9]. We also counted records, where the identification method was not stated. Moreover, we reported incorrect annotated sequence lengths. To this end, we measured sequence length and the number of ambiguous nucleotides with BioEdit Vers. 7.2.5 [21] in the fasta file employed and compared the annotated with the measured sequence length. However, we reduced the annotated sequence length by one bp, in case the length exceeded 657 bp, since the employed fasta file used sequences with maximum length of 657 bp due to the adjustment to the reading frame [9]. Furthermore, we registered records without sampling location registry conflicting with metadata requirements for submission [22]. We excluded the sequence with the process ID "AFTAC096-20" for further metadata analysis, because we received

the sequence via email and had no direct access to metadata on BOLD [9]. If the species assignment was registered in the "extra info" field and not in the "identification" field, the records were documented as "species annotated in the wrong field", as this blocks the sample identification by the BOLD query if these assignments represent the only representatives of their species.

Table 1. Classes of errors and gaps in BOLD metadata.

errors or gaps	class	description
incorrect sequence length	further metadata	The annotated sequence length of a record deviates from the measured sequence length
unknown identification method	further metadata	The identification method for a record is not mentioned in metadata
no information on sex	further metadata	The information on sex is not mentioned in metadata of a record
no country information	further metadata	The information on the sampling location is not given in metadata of a record
species annotated in wrong field	further metadata	The species assignment of a record is registered in the field "extra info" instead of the field "identification"
paraphyletic structure	BIN registry	The species identify represented by BINs is paraphyletic
entails 2 or more confirmed species	BIN registry	The BIN assigned to a record contains more than one species which contradicts the final species assignment of 9 [9]
assignment is conflicting	BIN registry	The BIN assignment of a record contradicts the BOLD OTU pipeline criteria [1]
due to unknown identification method	questionable species assignments	The species assignment of a record contradicts the species assignments predominant in a species clade. If the identification was COI-based this information should be removed
due to misidentification or species introgression	questionable species assignments	The species assignment stands in contradiction to the species assignments predominant in a clade. If the identification was due to human failures it had to be removed
missing species assignment	incorrect taxonomical assignment	The record is assigned to a BIN, but not to a species, although a COI-based identification is possible
outdated COI based identification	incorrect taxonomical assignment	There is a species assignment of a record relying on a COI-based identification, although there are contradictory species assignments in the assigned BIN
genus name is subgenus	incorrect taxonomical assignment	The subgenus name was applied as genus name of a record
outdated species name	incorrect taxonomical assignment	The assigned species name of a record is outdated
incorrect genus	incorrect taxonomical assignment	The record was wrongly assigned to the genus *Tachina*
incorrect species	incorrect taxonomical assignment	The record was assigned to the wrong *Tachina* species

Results

Accordance and discrepancies between final species assignments, BINs and ASAP partitions

For ten species identities, involving five described species, one cryptic species and four OTU species assignments, we found complete accordance between BIN, ASAP partitions, and final species assignment, forming monophyletic clades. In one instance, BIN BOLD:ACB0179 did not align with the final species assignment. RESL grouped *Tachina ursina* Meigen, 1824, sequences and a *Tachina nigrohirta* (Stein, 1924) sequence into the same BIN. However, the *Tachina nigrohirta* sequence was too short (<500 bp) to be considered by ASAP. Consequently, ASAP did not evaluate this sequence, but RESL still assigned it to BOLD:ACB0179, contradicting OTU pipeline criteria. The final species assignment, however, recognized *Tachina nigrohirta* as distinct from *Tachina ursina*, as *Wolbachia*-mediated species introgression or incomplete lineage sorting of closely related species could not be excluded [9]. For three single-sequence clades representing two described species *Tachina magna* (Giglio-Tos, 1890), *Tachina praeceps* Meigen, 1824 and one sequence assigned to the OTU name *Tachina* (Eudoromyia) sp.1, species assignments could not be confirmed by RESL and ASAP algorithms, because the sequences were too short.

Six species identities were confirmed by monophyletic clades and the ASAP partition, but BINs did not match these species identities. Specifically, the RESL algorithm was incapable to separate monophyletic branches representing *Tachina mongolica* (Zimin, 1935) and *Tachina ferox* Panzer, 1809, grouping them into a single clade corresponding to BIN BOLD:AAK1389 (Fig 1). Likewise, the species identities of closely related taxa *Tachina latifacies* (Tothill, 1924), *Tachina brevirostris* (Tothill, 1924) and *Tachina* (*Nowickia*) sp.2 were assigned to a single BIN, BIN BOLD:ABZ6159. For *Tachina algens* Wiedemann, 1830 two BINs (BOLD:ACE3327; BOLD:AAH6662) suggested putative cryptic species but

ASAP and final species assignments indicated only one species. The BIN BOLD:ACE3327 represented a paraphyletic group since it had a common ancestor, but did not include BIN BOLD:AAF6662. (Fig 1).

In five cases, species identities indicated by ASAP partitions and BINs were consistent. However, the final species assignments differed because revisions based on the phylogenetic relationships of closely related species were not performed [9]. This decision was made due to potential species introgression or incomplete lineage sorting among these species. Notably, within the subgenus *Eudoromyia*, *Tachina fera* (Zetterstedt, 1844) and *Tachina magnicornis* (Zetterstedt, 1844) were assigned to three putative species identities as detected by ASAP partitions and BINs. However, BIN BOLD:AAN9512 and the corresponding ASAP partition differed by one record, as ASAP ignored a sequence shorter than 500 bp, while RESL incorrectly included it. Therefore, the BIN included the record of *Tachina casta* (Rondani, 1859) rendering the putative species identity represented by the BIN polyphyletic.

Additionally, the monophyletic clade primarily containing *Tachina magnicornis* species assignments encompassed two ASAP partitions and the corresponding BINs BOLD:AAN9512 and BOLD:AEY0598 and contained the three species assignments, *Tachina fera*, *Tachina* nr. *magnicornis* and *Tachina corsicana* (Villeneuve, 1931). However, sequences allocated to two of the three species assignments were too short to be taken into account by ASAP and were allocated to BINs contradicting BOLD OTU pipeline quality criteria. The third OTU species *Tachina* nr. *magnicornis*, was indistinguishable from *Tachina magnicornis* and *Tachina fera* likely due to incomplete lineage sorting. Similarly, the RESL and ASAP algorithms failed to distinguish between the clade assigned to *Tachina marklini crypt 1* and *Tachina alpina* (Zetterstedt, 1849) (Fig 1). Furthermore, the clade predominantly composed of *Tachina lurida* (Fabricius, 1781) sequences additionally contained one sequence assigned to *Tachina ursina* (Meigen, 1824).

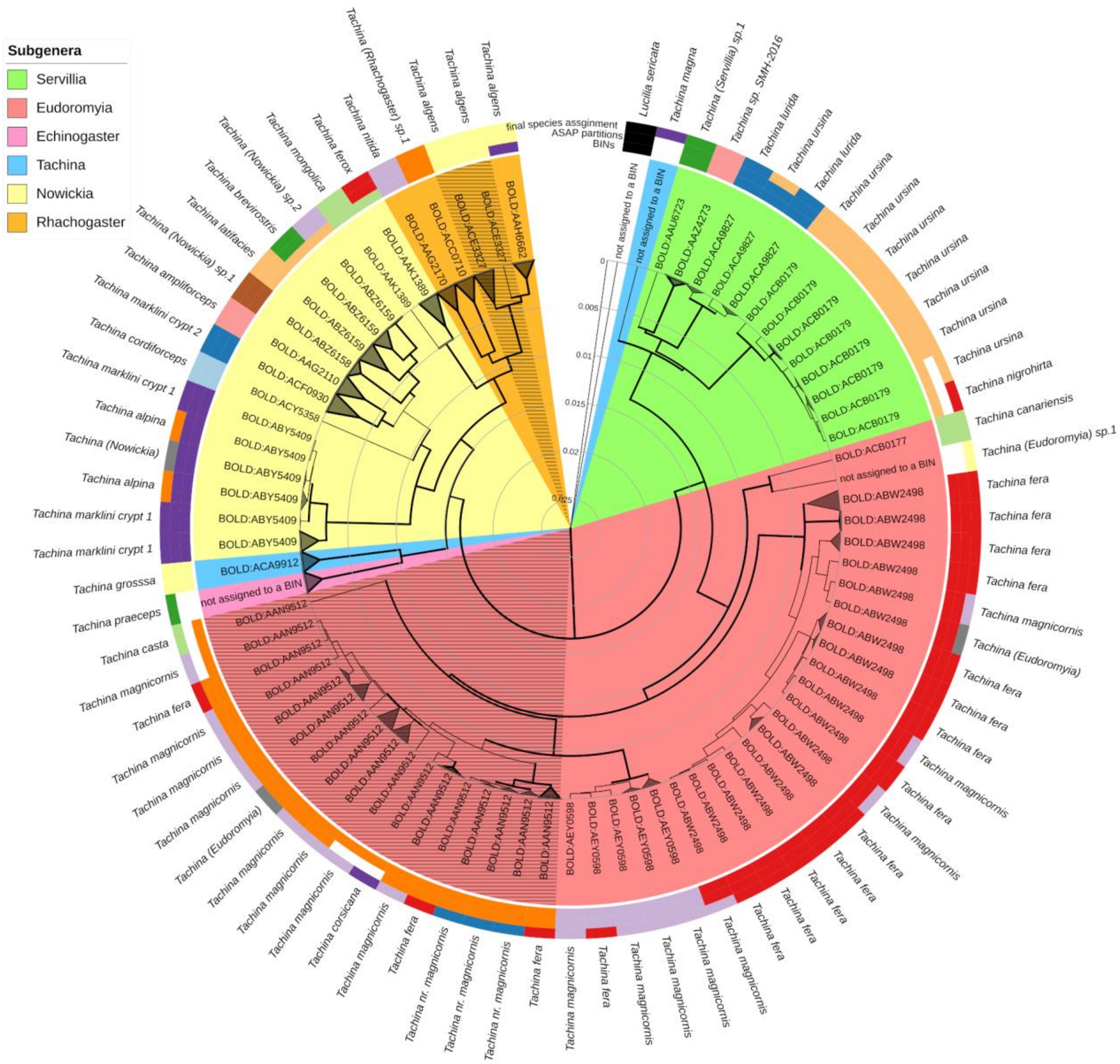

Fig 1. **Comparison of species identities detected by RESL, ASAP and clades containing final species assignments [9].** The original tree [9] was modified by adding BIN partitions and collapsing clades to the respective BIN partitions and hatching areas indicating non-monophyletic BIN species identities formatted by the RESL algorithm. The BINs, ASAP partitions and final species assignments are differentiated within subgenera by the inner, middle and outer colour strips. White stripes indicate that an assignment to a species identity was not possible. Colours were used once within one subgenus.

BOLD query results

In most cases, the results of a default BOLD v4 query corresponded to the BINs assigned to query sequences. However, in some cases no match to species could be achieved. This was the case when the query sequences were assigned to a BIN without a species assignment, when the species name was annotated in the wrong field or when records were assigned to a species and but not to a BIN (Table S2).

Moreover, in some cases, the results did not correspond to species assignments allocated to the BIN of the query sequence (Table 2). For *Tachina marklini* ~~(Zetterstedt, 1838)~~ sequences assigned to the BIN BOLD:ACF0930, which only incorporates species assignments of *Tachina marklini*, the species *Tachina ampliforceps* (Rowe, 1931) was also suggested in the results. Vice versa, for sequences allocated to the species name *Tachina ampliforceps* and assigned to BIN BOLD:AAG2110, additionally *Tachina marklini* was suggested as possible species match (Table 2).

We found a further discrepancy between the BOLD query results and the assigned BIN of the query sequences for sequences assigned to BIN BOLD:AEY0598. This BIN comprised the species names *Tachina magnicornis*, *Tachina fera* and the outdated species name [23] *Tachina nupta* (Zetterstedt, 1844). However, the query results yielded the species names *Tachina magnicornis*, *Tachina fera* and *Tachina* nr. *magnicornis*. Hence, the record assigned to *Tachina nupta* was disregarded. *Tachina* nr. *magnicornis* was exclusively allocated to BIN BOLD:AAN9512 while *Tachina magnicornis* and *Tachina fera* were allocated to BIN BOLD:AEY0598 and BIN BOLD:AAN9512. Therefore, the BOLD query with sequences assigned to BIN BOLD:AEY0598 triggered partially species assignments of BIN BOLD:AAN9512 (Table 2).

For sequences assigned to BIN BOLD:AAN9512, we also found differences between query results and species assignments of the BIN. For sequences assigned to *Tachina magnicornis*, *Tachina corsicana*, *Tachina fera* and *Tachina* nr. *magnicornis*, matches to *Tachina magnicornis*, *Tachina fera*, *Tachina* nr. *magnicornis* were found, while matches to *Tachina corsicana* and *Tachina casta* not corresponding to BOLD OTU pipeline criteria (sequence length < 500 bp) were not detected (Table 2). Moreover, the BOLD query with the sequence assigned to *Tachina casta* did result in no match. Merely, sequences assigned to the species *Tachina fera* were reported as nearest reference sequence. For BIN BOLD:ACB0179, which is assigned to the species *Tachina ursina* and *Tachina nigrohirta*, query sequences of both species yielded only *Tachina ursina* as species match and ignored the record assigned to *Tachina nigrohirta* not meeting BOLD OTU pipeline criteria (Table 2). Moreover, the BOLD

query with the “Public Record Barcode Database” as reference library resulted in a match with the species *Tachina nitida* assigned to BIN BOLD:AAG2170.

Table 2. Selection of BOLD query results deviating from reference BINs. A complete overview for BOLD queries can be found in the supplement (Table S2).

Annotated species		*Tachina marklini*	*Tachina ampliforceps*	*Tachina magnicornis*	*Tachina magnicornis*	*Tachina casta*	*Tachina corsicana*	*Tachina nigrohirta*	*Tachina ursina*
Process ID of query sequences		CNCDT783-12	BBDCP692-10	NLDIP289-12	BGTAC045-10	GBDP9352-10	GBDP9354-10	GBDP9356-10	TACFI100-12
BOLD OTU pipeline quality criteria	sequence length > 500 bp	fulfilled	fulfilled	fulfilled	fulfilled	not fulfilled	not fulfilled	not fulfilled	fulfilled
	> 1 % ambiguous nucleotides	fulfilled	fulfilled	fulfilled	fulfilled	fulfilled	fulfilled	fulfilled	fulfilled
Assigned BIN		BOLD:ACF0930	BOLD:AAG2110	BOLD:AEY0598	BOLD:AAN9512	BOLD:AAN9512	BOLD:AAN9512	BOLD:ACB0179	BOLD:ACB0179
Species assigned to BIN		*Tachina marklini*	*Tachina ampliforceps*	*Tachina magnicornis*<br>*Tachina fera*<br>*Tachina nupta*	*Tachina magnicornis*<br>*Tachina fera*<br>*Tachina* nr. *magnicornis*<br>*Tachina corsicana*<br>*Tachina casta*	*Tachina magnicornis*<br>*Tachina fera*<br>*Tachina* nr. *magnicornis*<br>*Tachina corsicana*<br>*Tachina casta*	*Tachina magnicornis*<br>*Tachina fera*<br>*Tachina* nr. *magnicornis*<br>*Tachina corsicana*<br>*Tachina casta*	*Tachina ursina*<br>*Tachina nigrohirta*	*Tachina ursina*<br>*Tachina nigrohirta*
Species matched in BOLD query		*Tachina marklini*<br>*Tachina ampliforceps*	*Tachina ampliforceps*<br>*Tachina marklini*	*Tachina magnicornis*<br>*Tachina fera*<br>*Tachina* nr. *magnicornis*	*Tachina magnicornis*<br>*Tachina fera*<br>*Tachina* nr. *magnicornis*	no species match	*Tachina magnicornis*<br>*Tachina fera*<br>*Tachina* nr. *magnicornis*	*Tachina ursina*	*Tachina ursina*
Discrepancy between BOLD query results and assigned BIN	missing			*Tachina nupta*	*Tachina corsicana*<br>*Tachina casta*	all species	*Tachina corsicana*<br>*Tachina casta*	*Tachina nigrohirta*	*Tachina nigrohirta*
	additional	*Tachina ampliforceps*	*Tachina marklini*	*Tachina* nr. *magnicornis*					

Publication state

For 22 annotated species names and 376 published records, only three records (0.8 % of all published records with species assignment) were available with a documented morphological identification in metadata. Nonetheless, based on personal communication [9], additional 12.8 % of samples were identified morphologically in published records. In contrast, for private records over 55% of species assignments relied on morphological identifications and 75%, when we consider information we received via email (personal communication). Morphological identifications were also conducted for more than half of the entries published after our request. Considering all sequences with species assignment, more than a quarter of all records were identified morphologically, 61.7 % were identified solely based on COI-5P sequences and for 12.3 % the identification method was unknown (Figure 2).

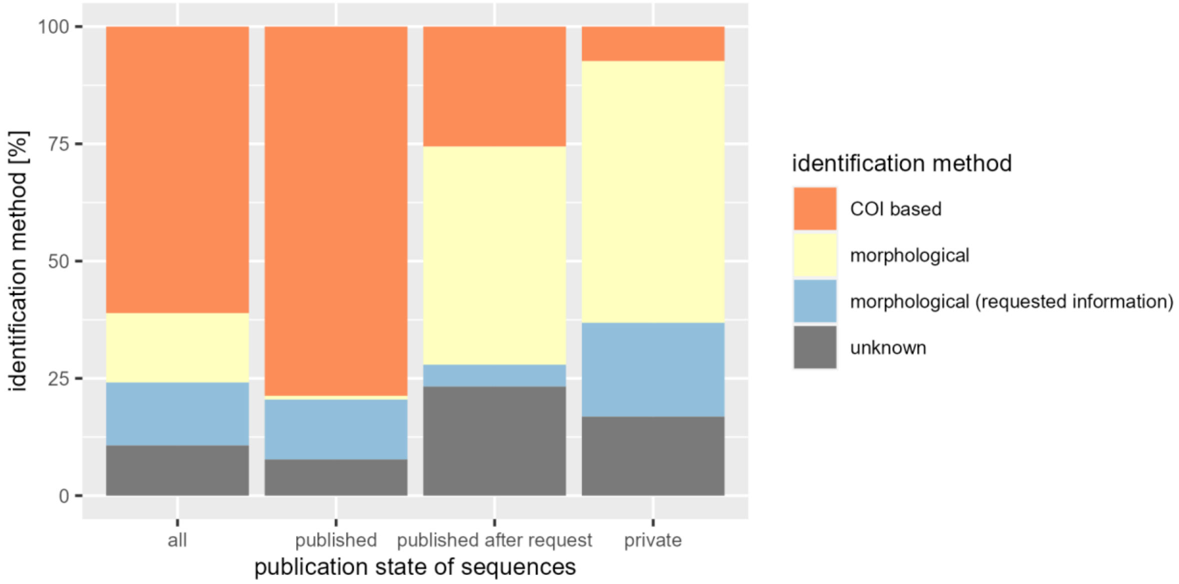

Figure 2. Publication state and identification method of sequences assigned to species.

Errors and lacks in BOLD annotations

Based on metadata [9], we detected incorrect taxonomical assignments. Nine records were wrongly assigned to the genus *Tachina* or to the incorrect species. For 17 sequences the subgenus name *Nowickia* was annotated as genus name and six sequences were assigned to junior synonyms. Additionally, for 30 sequences, the COI-based identification was outdated and the taxonomical

assignment should be removed. Vice versa, species assignments for 133 sequences were missing, although a COI-based identification would have been possible based on the dataset. However, for 32 of 133 records the species assignment was only possible based on the private annotations combined with ASAP partitions.

Moreover, we found records with “questionable species assignments”. We identified 11 records in data with species assignments that were inconsistent with most species assignments in the species clades. Furthermore, there were 27 records, where the identification method was unknown and the species assignment would have to be removed, in case the identification method was COI-based, as they were allocated to BINs with conflicting species assignments based on morphological identifications.

Beyond that, we spotted questionable BIN registries. We detected 19 sequences with BIN assignments conflicting with OTU pipeline criteria [1], because sequences were shorter than 500 bp or the frequency of ambiguous nucleotides was greater than one percent. Additionally, we registered 105 records in BINs, that each included at least two described species or species identities. Additionally, we counted 230 records assigned to paraphyletic putative cryptic species identities represented by the BINs BOLD:ACE3327 comprising a portion of *Tachina algens* records and BOLD:AAN9512 including records assigned to *Tachina magnicornis*, *Tachina fera*, *Tachina corsicana* and *Tachina casta* (Fig 1).

Furthermore, we detected false registrations and gaps in further metadata. For 340 records, the annotated sequence length was 1- 377 bp longer than the measured sequence length, for 66 records the sex was not specified, although a morphological identification was conducted (**Figure 3**) and for 90 records the identification method was not documented in metadata. Furthermore, the country was not specified for 18 records, which is contrary to the submission requirements. According to annotations, these records were mined from GenBank. Furthermore, for 14 records the species name was annotated wrongly in the field “extra info” instead the field “identification” (Figure 3). This applied to all records to which of the species *Tachina latifacies* and *Tachina brevirostris* were assigned in metadata.

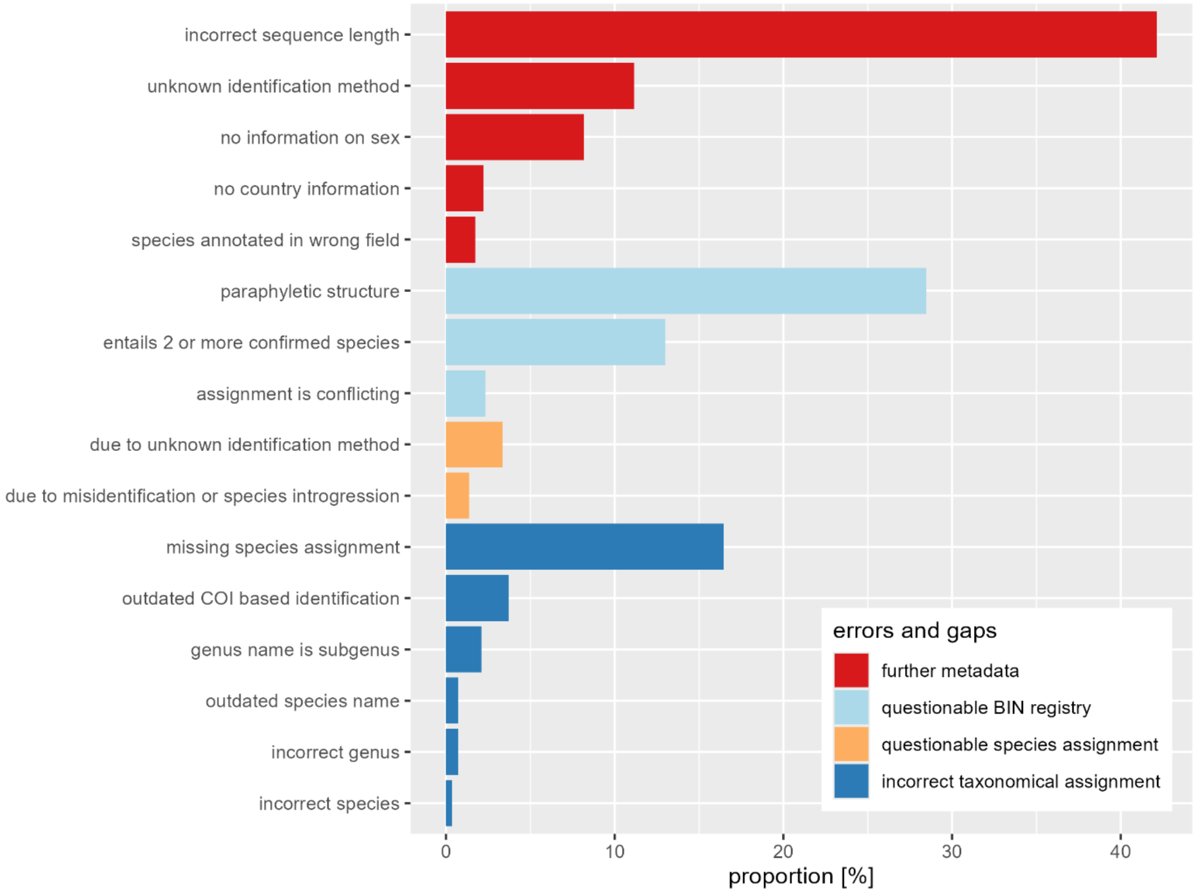

Figure 3. Proportion of errors and gaps in BOLD annotations of *Tachina* records (n = 812) obtained from BOLD.

Discussion

Our analyses revealed weaknesses in the employed species determination algorithm RESL, in the BOLD ID engine, BIN registry, data submission, and post-submission processes, promoting errors and gaps in metadata. In particular, the communication that private sequences can impact the public database is lacking.

ASAP yields more reliable species assignments than RESL

We found that both RESL [1] and ASAP algorithms [13] yielded results partly inconsistent with the final species assignments [9]. However, ASAP was superior in species determination. For example, it was capable to separate the group representing BIN BOLD:ABZ6159 into three species identities including *Tachina latifacies*, which had no barcoding gap. Similarly, ASAP split BIN BOLD:AAK1389 into two species, where *Tachina ferox* showed a remarkably small barcoding gap of 0.01 % of genetic divergence

[9]. Consequently, the species *Tachina latifacies* and *Tachina ferox* represent cases where lineage sorting is completed but a barcoding gap has not yet formed.

Furthermore, unlike RESL, ASAP did not separate *Tachina algens* records into two partitions, instead it grouped correctly all *Tachina algens* records into a single partition without a barcoding gap that matches the final species assignments. *Tachina algens* might represent an evolutionarily old species, where the accumulation of mutations over time has resulted in high intraspecific divergence, especially in cases of secondary contact between phylogeographical lineages [24]. In such situations, the genetic distance between some genotypes within the species can exceed the average distance to other closely related species. Consequently, it appears conceivable that interspecific genetic distances to other closely related species might be lower than the highest intraspecific distance. Apparently, in addition to the probability of barcoding gap width, ASAP considers the probability of partitions based on hierarchical clustering. Both probabilities are evaluated as *p* values for each partition. The average of both values is termed the ASAP score, with the lowest ASAP score corresponding to the most probable rank [13]. Therefore, ASAP enables the species distinction by introducing a second metric, allowing the concept of the DNA barcoding gap to be neglected where necessary.

However, the separation of a species clade dominated by *Tachina magnicornis* into two species partitions (corresponding to the BINs BOLD:AAN9512 and BOLD:AEY0598) by the ASAP and RESL algorithms was not supported by the final species assignments [9]. The observed polyphyly in BIN BOLD:AAN9512 was attributable to the sequence of *Tachina casta* that was shorter than 500 bp. Consequently, the BIN registration conflicted with the BOLD OTU pipeline criteria [1] and ASAP excluded the sequence from the partitioning process. As a result, the corresponding ASAP partition was monophyletic. This raises the question of whether ASAP would have also produced a polyphyletic species partition had the *Tachina casta* sequence been at least 500 bp in length, a question that remains unresolved. Additional partitions where both algorithms deviated from the final species assignments are likely due to insufficient resolution of the gene locus [9].

Among the concerned BINs, at least 85 % of the records were covered by our dataset and all contradictory species assignments in BINs were represented. Consequently, we consider it unlikely, that partitioning would have changed by ASAP if the remaining sequences had been added. Furthermore, we point out that the accordance between final species assignments and ASAP partitions is not due to circular reasons. Most species assignments, where RESL was unable to separate the species, were already specified in metadata and subsequently confirmed by ASAP. There was only one case where records were assigned to *Tachina* (*Nowickia*) sp.2, based on ASAP, which was not reflected in the metadata [9].

Therefore, we conclude, where COI-based separation was not restricted by the gene locus' weaknesses itself, the ASAP algorithm outperforms RESL in *Tachina* species determination, This improved performance is likely transferable to other arthropod groups, especially where high intraspecific genetic distances might exceed the average interspecific genetic distance, such as other Diptera [24], Miridae (Hemiptera) [25], Collembola or Oribatida [26]. Since ASAP considers the barcoding gap while incorporating additional parameters, we believe its performance in arthropods groups with distinct barcoding gap is unlikely to be inferior to RESL's. Direct comparison of RESL and ASAP across other arthropod groups may be challenging due to restricted access to private data on BOLD [9]. Consequently, we advocate replacing the RESL algorithm with another species determination algorithm (e.g., ASAP) that includes at least one additional parameter beyond the DNA barcoding gap to generate BINs in BOLD. Rather, previous work already predicted that analytic tools superior to RESL would be available for delimiting species boundaries from sequence data [1]. The suggestion of Meier et al. (2022) employing additional clustering algorithms might be expedient. However, we point out that the employment of additional algorithms results in an increased demand of computing capacity and could exceed BOLD servers' performance.

Amendment of the results page

Species suggested by the BOLD v4 ID engine deviated in some cases from reference BINs. Additionally, we found, that the BOLD v4 ID engine using the "Public Record Barcode Database", listed matched

species even when there was no support from records with morphological identification. We also found that records assigned to BINs without species assignments do not yield results with the BOLD v4 ID engine.

When query sequences triggered species names in query results conflicting with species of the BIN allocated to the query sequence, we surmised that the BIN assignment history of sequences played a role. Indeed, the delta view, a BOLD feature that provides information on the records' history, confirmed that the divergent species assignments belonged to BINs to which the query sequences were once assigned. The observed inconsistency of BOLD v4 ID engine default mode results could be due to a temporal lag between downloadable metadata and metadata employed by the BOLD v4 ID engine. The BOLD v4 ID engine's desirable exclusion of records that violate BOLD OTU pipeline criteria [1] could also be a result of a temporal lag. However, in case this temporal gap exists, we recommend closing it.

The listed species triggered by the BOLD query option, relying on the "Public Records Barcode Database", confirm that references assigned to morphological identifications are not required, as such records were not present in the reference library. Consequently, the species assigned to the record that allowed the triggering of BIN BOLD:AAG2170 was identified based on a reference sequence that was once assigned COI-based. Therefore, the reference sequence for this COI-based identifications must have relied on private data in the absence of other published sequences and the suggested higher degree of data curation of such a BOLD query is not necessarily given. However, we note that a BOLD query based on the "Public Record Barcode Database" with a *Tachina* sequence probably had resulted in no species match, as there were only three records identified morphologically to the species in published data, when we downloaded published sequences.

Another issue is that BINs without species assignment do not yield any matches when sequences assigned to these BINs are entered into the BOLD v4 ID engine. Hence, results suggest a lack of reference sequences. Furthermore, matched BINs are exclusively linked to the result's webpage in case there is only one possible species match. Otherwise, possible species matches are listed without

naming the BIN containing the reference records. Consequently, we advocate for a more transparent and comprehensive presentation of results. We recommend listing the matched BIN, all species assigned to this BIN and further BINs containing species assignments of the matched BIN as BOLD query result. Additionally, we suggest to list species names contained in one BIN and sharing the same epithet in one line indicating that species names are synonyms. In addition, it is useful to indicate the number of the morphologically determined specimens for the listed species names. Moreover, for the function displaying a neighbour joining tree, we recommend annotating the BINs and considering sequences representing all species of the genera or subgenera contained in the matched BIN.

The realization of the recommendations would provide an initial overview on tentative cryptic species and how contradictory species assignments supported by morphological identifications are distributed in the BINs. Thus, even if a species introgression exists, this gives the user an impression of the probability that their sequences correspond to one of both species. Ideally, results for BINs without species assignments would also be displayed, since the user receives information that a BIN was matched, even if there was no species match.

The new BOLD v5 ID engine partially addresses the reported issues. Firstly, the option to employ the public database is now the default mode. Secondly, a species match based on the BIN is only displayed on the results page if the matched BIN contains one species assignment. Otherwise, solely the genus name is called. However, the Process ID and BIN have been added as information for the list of the closest records. This allows to trace, which BIN was matched in case it does not contain any species assignments, and additionally provides the information on which species are assigned to the matched BIN. Unfortunately, the option to generate a neighbour joining tree has been removed. Likewise, for the BOLD v5 ID engine the option “Animal Library (Public + Private)” corresponding to the default mode of the BOLD v4 ID engine, publication state of the closest records is no longer displayed. Furthermore, we note that the orientation of entered sequences is adapted, when entering a sequence with reverse orientation into the BOLD v5 ID engine. However, it is not displayed that the sequence has a reverse orientation.

Sequence submission process optimization by focusing on identification method and introducing a third data state

We consider several errors and gaps in annotations to be related with the submission process. Especially, we consider incorrect species assignments are at least partially the result of a lack of communication by BOLD with users with regard to published and private sequences. Although the BOLD manual on the BOLD v4 ID engine indicates that private entries are also included in the results of default BOLD database queries [27], this is not communicated in the chapter on the submission process or during the submission process. The fact that private records with morphological references are more abundant compared to published records indicates users' reluctance to publish these records, possibly are due to uncertainties in morphological identifications. Consequently, records associated with uncertain morphological identifications could be uploaded to BOLD, which affect the default mode of the BOLD v4 ID engine. As the default mode was changed from BOLDv4 (private and public data) to the BOLD v5 ID engine (public data), the issue is expected to be mitigated, but it is still possible to conduct a COI-based identification using one of the other BOLD query modes (Table 3).

Nevertheless, we suggest clearly communicating during record submission that private records can be employed by the BOLD v5 ID engine by two search options (Animal-species-level library, public + private; Animal library, public + private). Furthermore, we recommend the introduction of a third data state, aside from private and published records, which might be labelled as preliminary submitted records. In this case, data would not be considered for BIN formation by the employed species determination algorithm (Table 3). However, an automatized BOLD query could provide information on possible species and BIN matches of the submitted sequences and on the number of records per species associated with a morphological identification.

Moreover, we recommend amending data sheets for the record submission. The creation of a separate section for the neglected issue of identification might be sensible in order to assess the species assignment procedure from a third person's perspective. Furthermore, we argue for mandatory

documentation of the identification method. We also believe that registering sex information during submission should be encouraged, at least for adult specimens in arthropod groups where species identification requires predominantly sex determination, such as several dipteran families, for instance the Tachinidae or Sarcophagidae [19,28] or for groups such as hymenopteran Symphyta, where the sex is often obvious or easily determined (Table 3).

Furthermore, we consider providing default options for the identification method field like "COI-based", "morphological" and "others" and possibly a second non-mandatory field to specify the chosen identification method. We support the recommendation of previous work [12] to introduce two additional mandatory annotation fields: the identification reference (such as species keys) and the author of the original species description, in accordance with the International Code of Zoological Nomenclature [29]. These measures would help avoid synonyms, facilitate species identification in BOLD, and enable the tracing of morphological identifications. In case morphological identification is uncertain, there should be an extra field to document possible species without assigning an uncertain species name in the "identification" field.

Moreover, we propose an optional subgenus field for the taxonomy section in combination with a second mandatory field for the author of first subgenus description, in case subgenus assignment is performed. This enables more detailed information about the taxonomic position, in order to specify uncertain morphological identifications and to detect taxonomically incorrect species assignments within genera [9]. Another issue related to taxonomy is the utilization of OTU names in metadata, whereby the reason for their employment often remains unclear as there is a lack of clear rules for the use of OTU names. We suspect that OTU names were attributed to enable their identification via BOLD queries. However, since the new BOLD v5 ID engine also shows BINs without species assignments on the results page the employment of OTU names could be reduced prospectively. To address the database errors related to the species assignment in the non-prescribed field "extra info", we think current submission rules are sufficient, provided they are rigorously applied (Table 3).

Automatic generation of metadata and integration of a Bayesian tool evaluating questionable species assignments post submission

Besides metadata created by the submitter during the submission process, there are automatically generated metadata such as BIN assignment or sequence length. However, it appears, that this has not always been the case, because there are annotated sequence lengths, which are incorrect and might be due to older sequence uploads. Especially for sequences mined from GenBank, an additional control for annotated sequence length presumably is missing. Therefore, we recommend evaluating and revising older metadata. Furthermore, we think additional field registrations, such as the date of sequence uploads, the date of species assignment and the date of BIN assignment, could be generated automatically to provide traceability of record history. These are exclusively accessible in the delta view of single records, and obtaining theses data for multiple records is laborious [10]. We consider they could be automatically allocated to metadata. In addition, the data in delta view is only accessible for published records. We therefore recommend developing a delta view on BINs (Table 3). This way, the date of BIN formation and the assignment of species names could be retraced rapidly and independently of record publication state.

The missing species assignments could be due to the situation that the BINs to which the newly uploaded sequences were allocated at the time of uploading were not yet assigned to species. We recommend automatic species assignment if the BIN does not contain any contradictory species assignments. In case of BINs without species assignments in BOLD metadata, we recommend an automatized message via the workbench to data managers of respective sequences with the request for the possibility of a morphological sample identification (Table 3). However, we recommend asking responsible data managers only once per specimen, as repeated message requests should not pressure

the identifier to assign the species, although the morphological identification is related to uncertainties.

In case a new record has been uploaded with a species assignment that causes contradictory information in BINs, action is required by BOLD. We recommend verifying whether the new sequence leads to a non-monophyletic arrangement of the old sequences associated with morphological identifications within the BIN for example by integrating an R protocol to BOLD. First, the protocol could test, if there is a sequence identical to the newly uploaded sequence. The sequence match and the conflicting species assignment of the new sequence to one of the existing sequences would impede monophyly of sequences already assigned to the BIN. In case there is no identical sequence, we suggest initiating an automated Bayesian phylogenetic analysis by employing records relying on a morphological identification. The required computation capacity could be limited by restricting the number of examined sequences, whereby the new sequence and most similar sequences with the older species assignment must be part of the analysed sequence group. The packages required for such a R protocol to align [30] and edit fasta files [31], to detect the best fitting substitution model [32] and to integrate substitution models into a Bayesian phylogenetic tree reconstruction [33] and to annotate resulting trees [34] are available. In case the protocol allocates all sequences with the old species assignment to a monophyletic branch appearing as a sister group of the newly submitted sequence and the respective node is supported by a high posterior value, this might be an additional supporting metric for a BIN split. If old species assignments are non-monophyletic, we recommend sending an automatized message to the data manager responsible for the new record causing contradictory species information within the BIN. The message might contain a checklist asking for possible reasons of misidentification. On the condition, this does not solve the issue, we recommend automatic deletion of the species assignment relying on COI-based identifications contained in the concerned BIN (Table 3).

In the context of incorrect taxonomical assignments, the detection of records incorrectly assigned to a genus could be a major challenge at post-submission processes. These records might be detectable

with reduced Bayesian analyses in case they are assigned to BINs where other records with different genus assignments are present. Otherwise, their detection will be challenging, although these records were easily detectable with the Bayesian analysis of the whole genus, as they were clustered outside the genus [9]. This process demands computation capacity that surely cannot be provided by BOLD servers for every sequence submission. Less complex tools like neighbour joining approaches might not be reliable enough, as they do not provide information on the probability for nodes [35].

Enforcement of OTU pipeline criteria and submission requirements

Furthermore, we found records assigned to BINs conflicting with sequence quality criteria of the BOLD OTU pipeline [1] and missing sample locations in BOLD annotations standing in contradiction to sequence submission requirements [22]. We encourage to investigate and resolve the origin of this type of database error and to enforce rules for submission and sequence BIN assignment (Table 3).

Table 3: **Recommendations to amend the BOLD engine.** Recommendations in bold text are considered in BOLD v5

Issues of BOLD v4 engine	Recommendation	Solution on BOLD v5
RESL algorithm underperforms compared to ASAP due to reliance solely on barcoding gap	Employ a more recent algorithm (e.g., ASAP) that uses at least one additional metric besides the barcoding gap	No
Species assignments for one BIN based on public data often lack morphological references	**List all species assigned to the matched BIN**, and display the number of morphologically identified records for each species assignment within the BIN as BOLD query results	Partial
BINs without species assignments cannot be matched	**Display the matched BIN even without species assignments as BOLD query results**	Yes
Default BOLD query mode is based on private and public references	**Change default mode to public references only**	Yes
Lack of communication that private data can affect public references	Improve transparency during data submission and clearly communicate the impact of private records on public records; introduce a third data state ("preliminary"); **set default mode to public references only**	Partial
Missing meta data on identification method, identification references, authority of first description, sex and subgenera	Revise submission datasheets: introduce mandatory, default options for identification method; mandatory field registrations for authority of first description, introduce a subgenus field, and encourage user to give information on sex where applicable	No
No clear guidance on the use of OTU names	Prohibit the use of OTU names, **as BINs without species assignments can now be matched**	Partial

Metadata of mined records from GenBank often have incorrect sequence length	Apply automated sequence length validation retroactively to older records, especially GenBank imports	No
Record history traceability requires manual inspection of delta view for public data	Automate record history in metadata (e.g., submission data, BIN assignment) and introduce a BIN-level delta view	No
Records in BINs without species assignments and no conflicting species assignments	Automatically assign species names if BINs contains no conflicting species assignments	No
Records in BINs with conflicting species assignments	Contact responsible data manager species assignment verification; implement an automated bioinformatics protocol to assess BIN phylogeny	No
COI-based identifications causing conflicting species assignments within a BIN	Remove COI-based species names from BINs where conflicts cannot be resolved	No
Records assigned to BINs that do not meet OTU pipeline quality criteria	Enforce compliance with OTU pipeline requirements (e.g., sequence length, sampling location)	No
Species matches do not correspond to current BIN or metadata	Investigate mismatches between BOLD ID engine outputs and downloadable metadata; improve synchronization between BOLD ID engine and downloadable metadata;	Unclear

Conclusion

Despite, the aforementioned problems in BOLD v4, we consider BOLD as the most important DNA barcoding reference database due to the unreached amount of COI-5P based records, a high coverage of metazoan species and a high reliability compared to other COI-5P libraries like GenBank [36]. However, there is a need to improve the BOLD engine in several aspects to avoid the accumulation of incorrect taxonomical assignments in BINs. This has a particular impact on BOLD query results and will give users the impression of decreasing the reliability of BOLD, as long as the discussed origin of described errors and gaps in metadata is not addressed. In BOLD v5 multiple identified systematic problems related to the employed species determination algorithm, BOLD submission process and post submission process will remain, as RESL still generates BINs and the submission and post-processes still rely on the BOLD v4 engine. In case of meta-barcoding studies, where biodiversity indices are calculated and compared based on the BINs, the current state of BOLD might be sufficient. However, studies using BOLD as reference library to identify the species' specific ecological features such as host or prey preferences might be related to uncertainties.

Acknowledgements

We are grateful all institutions, project leaders and responsible data managers of BOLD, who enabled this study by providing access to their data in the scope of our former research [9]. We acknowledge the German Barcode of Life project, the Centre of Biodiversity Genomics, the Canadian Collection of Insects, the Royal British Columbia Museum, the Natural History Collections of the University Museum of Bergen, the Norwegian Institute, the University of Oslo, the University of Oulu, the Bavarian State Collection for Zoology, and the Naturalis Biodiversity Center for giving us access to private data or publishing private records and for providing supplemental metadata.

Supporting Information

Table S1: Updated metadata (4th September 2023) of sequence data.

Table S2: BOLD query results

Author contributions:

Frederik Stein (Conceptualization [lead], Data analysis [lead], Methodology [lead], Visualization [lead], Writing—original draft [lead]), and Oliver Gailing (Supervision [lead], Writing—review & editing [lead], Conceptualization [supporting])

Benefits sharing: The employed data were curated in terms of enhancing meta data of BOLD and GenBank data as described in 9 [9]. All new data obtained in the previous study was based on sampling carried out in accordance with the regulations in force in the country concerned and fulfil the conditions for access to genetic resources laid down in the Nagoya Protocol.

Funding statement: The present study was part of a research network (Federal Ministry of Food and Agriculture/ FNR: Risk Management for Biotic Damage to Ensure Sustainable Forest Management (RiMa) – FKZ 22019814). Partial aspects were financed by the Federal Research Centre for Cultivated Plants (Julius Kuehn Institute, JKI).

Conflict of Interest Statement: The funders had no role in the design of the study; in the collection, analyses, or interpretation of data; in the writing of the manuscript; or in the decision to publish the results. The authors declare no conflicts of interest.

Ethics approval statement: Ethics approval was not required, as all data were compiled by data mining.

References

1. Ratnasingham S, Hebert PDN. A DNA-based registry for all animal species: the barcode index number (BIN) system. PLOS ONE. 2013; 8:e66213. doi: 10.1371/journal.pone.0066213 PMID: 23861743.

2. Virgilio M, Backeljau T, Nevado B, de Meyer M. Comparative performances of DNA barcoding across insect orders. BMC Bioinformatics. 2010; 11:206. doi: 10.1186/1471-2105-11-206.

3. Smith MA, Woodley NE, Janzen DH, Hallwachs W, Hebert PDN. DNA Barcodes reveal cryptic host-specificity within the presumed polyphagous members of a genus of parasitoid flies (Diptera: Tachinidae). PNAS. 2006; 103:3657–62. doi: 10.1073/pnas.0511318103.

4. Taddei A, Becker M, Berger B, Da Lio D, Feltgen S, König S, et al. Molecular identification of Anoplophora glabripennis (Coleoptera: Cerambycidae) and detection from frass samples based on real-time quantitative PCR. J Plant Dis Prot. 2021; 128:1587–601. doi: 10.1007/s41348-021-00501-7.

5. Pohjoismäki JLO, Kahanpää J, Mutanen M. DNA Barcodes for the Northern European Tachinid Flies (Diptera: Tachinidae). PLOS ONE. 2016; 11:e0164933. doi: 10.1371/journal.pone.0164933.

6. Smith MA, Bertrand C, Crosby K, Eveleigh ES, Fernandez-Triana J, Fisher BL, et al. *Wolbachia* and DNA barcoding insects: patterns, potential, and problems. PLOS ONE. 2012; 7:e36514. Epub 2012/05/02. doi: 10.1371/journal.pone.0036514 PMID: 22567162.

7. Jäckel R, Mora D, Dobler S. Evidence for selective sweeps by *Wolbachia* infections: phylogeny of *Altica* leaf beetles and their reproductive parasites. Mol Ecol. 2013; 22:4241–55. doi: 10.1111/mec.12389 PMID: 23927410.

8. Pentinsaari M, Ratnasingham S, Miller SE, Hebert PDN. BOLD and GenBank revisited - Do identification errors arise in the lab or in the sequence libraries. PLOS ONE. 2020; 15:e0231814. Epub 2020/04/16. doi: 10.1371/journal.pone.0231814 PMID: 32298363.

9. Stein F, Gailing O, Moura CCM. Curating BOLD records via Bayesian phylogenetic assignments enables harmonization of regional subgeneric classifications and cryptic species detection within the genus Tachina (Diptera: Tachinidae). Ann Entomol Soc Am. 2024:245-256. doi: 10.1093/aesa/saae018.

10. Meier R, Blaimer BB, Buenaventura E, Hartop E, Rintelen T von, Srivathsan A, et al. A re-analysis of the data in Sharkey et al.'s (2021) minimalist revision reveals that BINs do not deserve names, but BOLD Systems needs a stronger commitment to open science. Cladistics. 2022; 38:264–75. doi: 10.1111/cla.12489.

11. Sharkey MJ, Janzen DH, Hallwachs W, Chapman EG, Smith MA, Dapkey T, et al. Minimalist revision and description of 403 new species in 11 subfamilies of Costa Rican braconid parasitoid wasps, including host records for 219 species. Zookeys. 2021; 1013:1–665. doi: 10.3897/zookeys.1013.55600.

12. Collins RA, Cruickshank RH. The seven deadly sins of DNA barcoding. Mol Ecol Resour. 2013; 13:969–75. Epub 2012/12/27. doi: 10.1111/1755-0998.12046 PMID: 23280099.

13. Puillandre N, Brouillet S, Achaz G. ASAP: assemble species by automatic partitioning. Mol Ecol Resour. 2021; 21:609–20. doi: 10.1111/1755-0998.13281.

14. Ratnasingham S, Wei C, Chan D, Agda J, Agda J, Ballesteros-Mejia L, et al. Chapter 26 - BOLD v4: A Centralized Bioinformatics Platform for DNA-Based Biodiversity Data. In: DeSalle R, editor. DNA Barcoding - Methods and Protocols. New York: Humana Press; 2024.

15. R Core Team. R: A language and environment for statistical computing. R foundation for statistical computing, Vienna, Austria. 2023. Available from: https://www.R-project.org/.

16. Posit Team. RStudio: Integrated Development Environment for R. Posit Software. Boston: RStudio, PCB. 2023. Available from: http://www.posit.co/.

17. Wickham H, Averick M, Bryan J, Chang W, McGowan L, François R, et al. Welcome to the Tidyverse. JOSS. 2019; 4:1686. doi: 10.21105/joss.01686.

18. Letunic I, Bork P. Interactive tree of life (iTOL) v3: an online tool for the display and annotation of phylogenetic and other trees. Nucleic Acids Res. 2016; 44:W242-5. doi: 10.1093/nar/gkw290 PMID: 27095192.

19. Tschorsnig H-P, Herting B. Die Raupenfliegen (Diptera; Tachinidae) Mitteleuropas. Bestimmungstabellen und Angaben zur Verbreitung und Ökologie der einzelnen Arten. Stuttg Beitr Naturkd Ser A. 1994; 506:1-170. Available from: https://www.biodiversitylibrary.org/part/41664.

20. Novotná H, Vaňhara J, Tóthová A, Muráriková N, Bejdák P, Tkoè M, et al. Identification and taxonomy of the West Palaearctic species of *Tachina* Meigen (Diptera: Tachinidae) based on male terminalia and molecular analysis. Entomol Fenn. 2009; 20:139–69. doi: 10.33338/ef.84472.

21. Hall TA. BioEdit: A user- friendly biological sequence alignments editor and analysis program for Windows 95/98/NT. Nucl Acid S. 1999; 41:95–8. doi: 10.12691/ajmr-3-2-1.

22. Milton MA, Pierosii P, Ratnasingham S. BOLD systems - Barcode of Life Data Systems Handbook - version 3.6. Guelph; 2013.

23. Cerretti P. I Tachinidi della fauna italiana (Diptera Tachinidae) con chiave interattiva dei generi ovest-paleartici. Verona: Centro Nazionale Biodiversitá Forestale; 2010.

24. Morinière J, Balke M, Doczkal D, Geiger MF, Hardulak LA, Haszprunar G, et al. A DNA barcode library for 5,200 German flies and midges (Insecta: Diptera) and its implications for metabarcoding-based biomonitoring. Mol Ecol Resour. 2019; 19:900–28. Epub 2019/05/14. doi: 10.1111/1755-0998.13022 PMID: 30977972.

25. Kim J, Jung S. COI barcoding of plant bugs (Insecta: Hemiptera: Miridae). PeerJ. 2018; 6:e6070. Epub 2018/12/04. doi: 10.7717/peerj.6070 PMID: 30533322.

26. Le Cadre J, Klemp FL, Bálint M, Scheu S, Schaefer I. Applicability and perspectives for DNA barcoding of soil invertebrates. PeerJ. 2024; 12:e17709. doi: 10.7717/peerj.17709.

27. Milton MA, Rahulan M, Elliott TA, Ho C, Kleint-Steinke C, Manjunath R, et al. BOLD systems - Barcode of Life Data Systems Handbook - version 4.0. 2023 [updated February 2023; cited 28 Jan 2024]. Available from: https://bench.boldsystems.org/libhtml_v3/static/BOLD4_Handbook_FinalVersion_Feb2023.pdf.

28. Povolný D, Verves Y. The flesh-flies of central Europe (Insecta, Diptera, Sarcophagidae). München: Pfeil; 1997.

29. Ride WDL, Cogger HG, Dupuis C, Kraus O, Minelli A, Thompson FC, et al. International code of zoological nomenclature. 4th ed. London: International Trust for Zoological Nomenclature; 1999.

30. Edgar RC. MUSCLE: a multiple sequence alignment method with reduced time and space complexity. BMC Bioinformatics. 2004; 5:1–19. Epub 2004/08/19. doi: 10.1186/1471-2105-5-113 PMID: 15318951.

31. Revell LJ. phytools: an R package for phylogenetic comparative biology (and other things). Methods Ecol Evol. 2012; 3:217–23. doi: 10.1111/j.2041-210X.2011.00169.x.

32. Schliep KP. phangorn: phylogenetic analysis in R. Bioinformatics. 2011; 27:592–3. Epub 2010/12/17. doi: 10.1093/bioinformatics/btq706 PMID: 21169378.

33. Bilderbeek RJC, Etienne RS. babette: BEAUti 2, BEAST2 and Tracer for R. Methods Ecol Evol. 2018; 9:2034–40. doi: 10.1111/2041-210X.13032.

34. Xu S, Li L, Luo X, Chen M, Tang W, Zhan L, et al. Ggtree : A serialized data object for visualization of a phylogenetic tree and annotation data. iMeta. 2022; 1. doi: 10.1002/imt2.56.

35. Yang Z. Computational Molecular Evolution. Oxford, New York: Oxford University Press; 2006.

36. Baena-Bejarano N, Reina C, Martínez-Revelo DE, Medina CA, Tovar E, Uribe-Soto S, et al. Taxonomic identification accuracy from BOLD and GenBank databases using over a thousand

insect DNA barcodes from Colombia. PLOS ONE. 2023; 18:e0277379. Epub 2023/04/24. doi: 10.1371/journal.pone.0277379 PMID: 37093820.

Comparison of species identities detected by RESL, ASAP and clades containing final species assignments [9].

Click here to access/download;Figure;Fig1.png

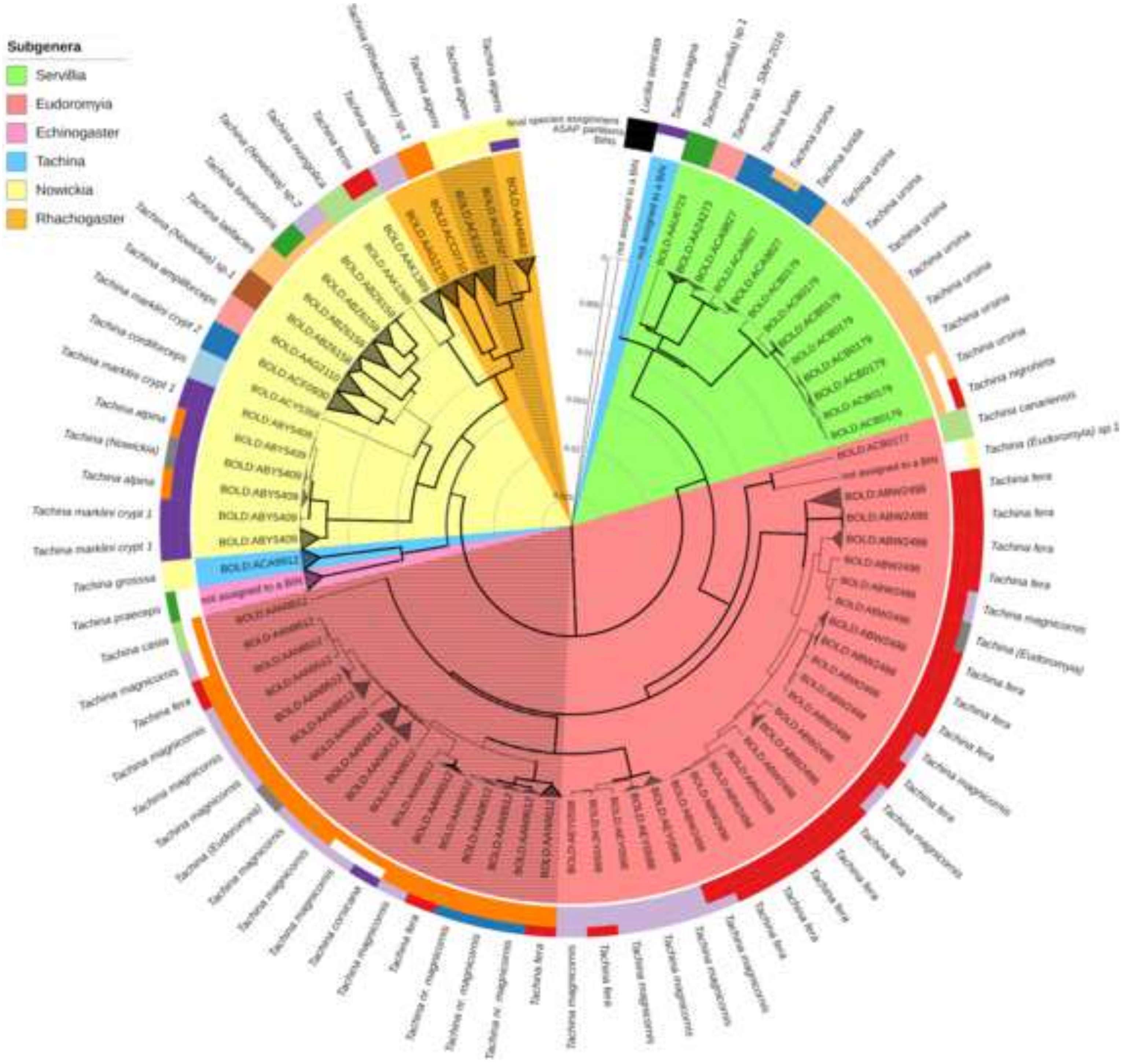

Publication state and identification method of sequences assigned to species.

Click here to access/download;Figure;Fig2.png

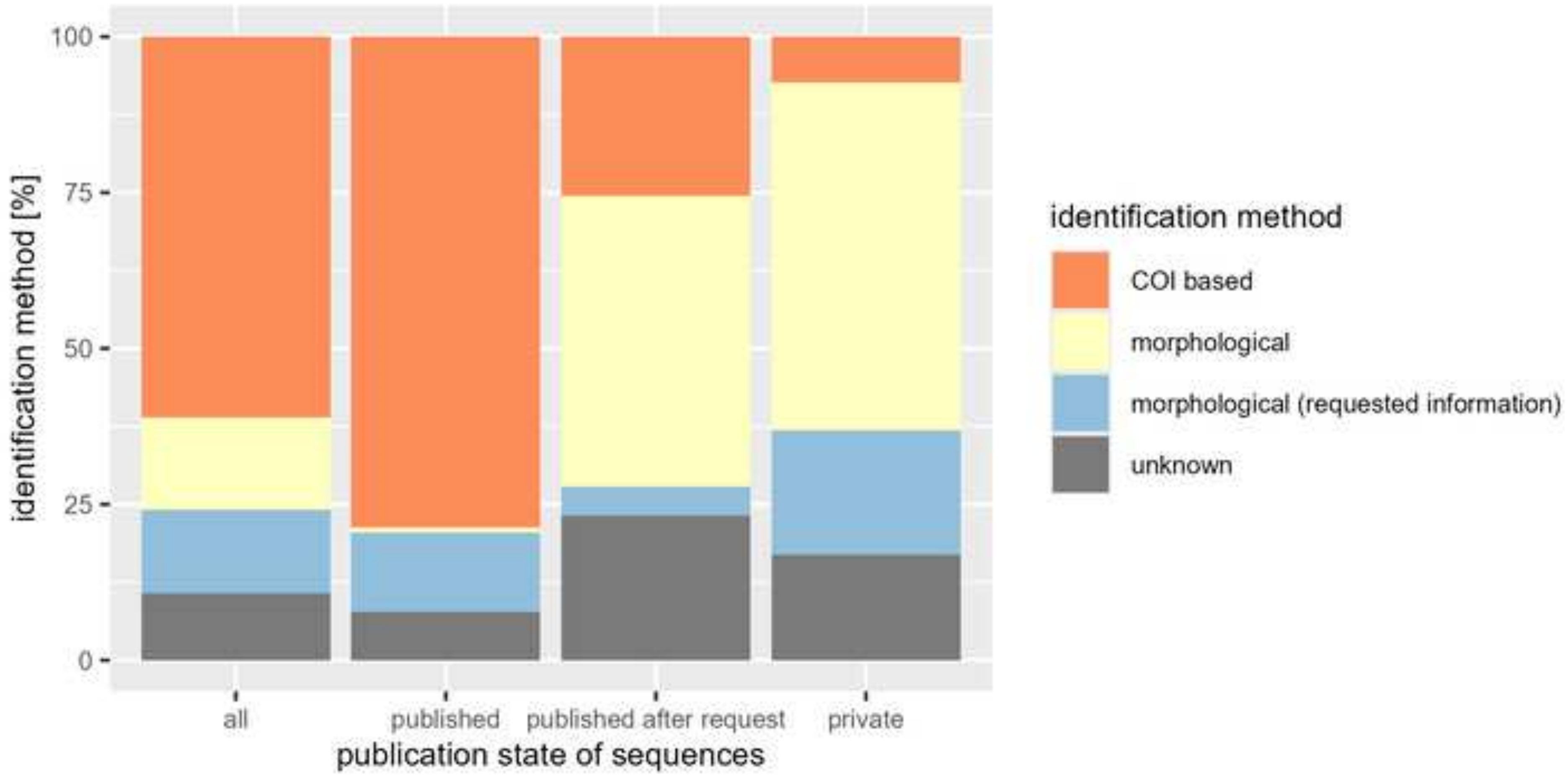

Proportion of errors and gaps in BOLD annotations of Tachina records (n = 812) obtained from BOLD.

Click here to access/download;Figure;Fig3.png

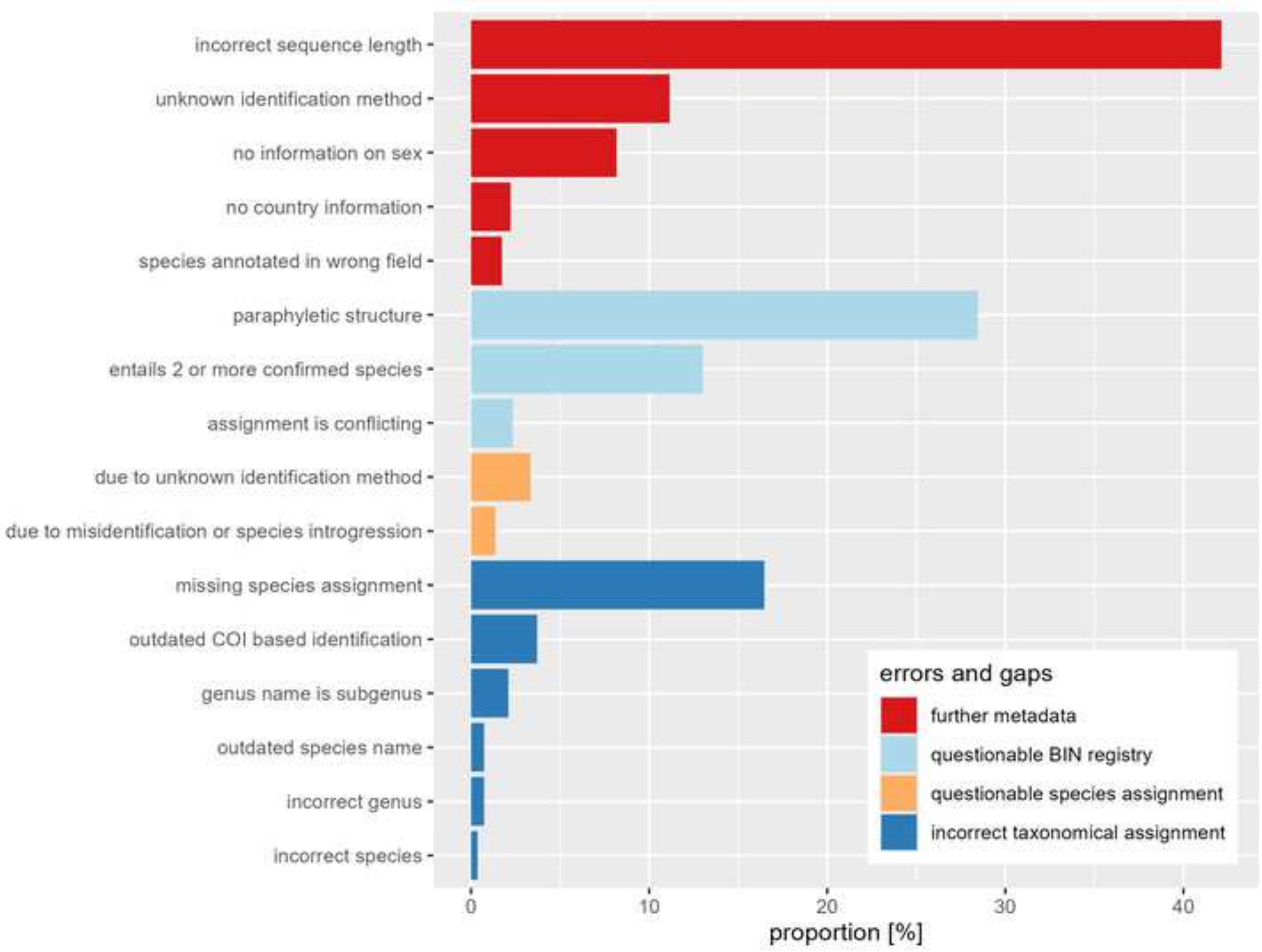

Click here to access/download
Supporting Information - Compressed/ZIP File Archive
Supporting Information.7z
